# Topical Approach to Delivering Targeted Therapies in Lymphedema Treatment: A Systematic Review

**DOI:** 10.7759/cureus.6269

**Published:** 2019-12-01

**Authors:** Antonio J Forte, Daniel Boczar, Maria T Huayllani, Sarah A McLaughlin, Sanjay Bagaria

**Affiliations:** 1 Plastic Surgery, Mayo Clinic Florida Robert D. and Patricia E. Kern Center for the Science of Health Care Delivery, Jacksonville, USA; 2 Surgery, Mayo Clinic Florida Robert D. and Patricia E. Kern Center for the Science of Health Care Delivery, Jacksonville, USA

**Keywords:** topical therapy, lymphedema, breast cancer lymphedema, vegf, vascular-endothelial growth factor, microsurgery, lymphatic surgery, pharmacologic treatment, upper extremity lymphedema, lower extremity lymphedema

## Abstract

It is estimated that 140 to 200 million people are affected by lymphedema worldwide. Many studies have proposed targeted therapies that can be delivered systemically or locally to treat lymphedema. Since lymphedema primarily affects the skin and subcutaneous tissues, topical approaches to therapy should be considered as an attractive proposition as they can avoid systemic complications. In light of this, we conducted a systematic review of publications that analyzed the use of topical approaches to delivering targeted therapies in the treatment of lymphedema. We hypothesized that topical approaches resulted in the satisfactory treatment of lymphedema. We conducted a systematic review of publications on PubMed. The main eligibility criterion was that the articles should primarily investigate the use of topical approaches to delivering targeted therapies in the treatment of lymphedema. Consequently, we excluded papers that investigated any other delivery approaches or medical conditions. Of the 174 potential studies found in the literature, six were found to fulfill our eligibility criteria. All these studies were experimental ones on small animals (mice). The authors generally proposed different types of therapies, which could be clustered into two main groups: 1) induction of lymphangiogenesis [vascular endothelial growth factor C (VEGF-C) hydrogel or fibroblast growth factor]; and 2) modulation of inflammation (tacrolimus or topical collagen gel or troxerutin-phosphatidylcholine). All studies presented positive outcomes, demonstrating that topical therapy is a promising route for delivering growth factors and anti-inflammatory agents in the treatment of lymphedema. However, studies were conducted under heterogeneous protocols, and the safe application of these therapies in humans has not been assessed. Further studies are necessary to confirm the benefits and safety of targeted topical therapy on patients with lymphedema.

## Introduction and background

Studies suggest that 140 to 200 million people are affected by lymphedema worldwide [1,2]. In developed nations, the most common cause of lymphedema is cancer treatment, affecting one in every six patients that undergo solid tumor resection [3]. Affected patients typically present with symptoms only months after oncologic treatment, and this indicates that the pathophysiology of this medical condition is promoted by an imbalance between lymphatic regeneration and tissue inflammation/fibrosis [4-6].

In light of the high prevalence of lymphedema and the lack of any curative approach to the disease, many studies have proposed targeted therapies in the treatment of lymphedema. The two most common rationale used in many studies is the induction of lymphangiogenesis and control of inflammation/fibrosis [7-12]. Studies have demonstrated that vascular endothelial growth factors (VEGFs) are able to ameliorate lymphedema on animal models [10,11]. However, the idea of adopting these targeted therapies in clinical practice remains controversial as there is potential for adverse outcomes such as the increased risk of metastasis among oncology patients [11].

Different delivery routes for targeted therapies have been proposed in the treatment of lymphedema, including oral, topical, and subcutaneous or intravenous injections. Since lymphedema primarily affects the skin and subcutaneous tissues, topical approaches could avoid systemic complications [8]. We conducted a systematic review of published studies that analyzed the use of topical approaches to delivering targeted therapies in the treatment of lymphedema. We hypothesized that a topical approach could lead to satisfactory treatment for lymphedema.

## Review

Materials and methods

Search Strategy

Two reviewers (D.B and M.T.H) conducted independent searches using the PubMed database without any timeframe limitations, initially through the title and abstract screen and then by full-text review. Disagreements regarding article identification and final selection for the inclusion of the literature were resolved by another reviewer (A.J.F). A search was done using the following keywords: (((Lymphedema) OR Breast Cancer Lymphedema)) AND ((((((Topical) OR Cream) OR Gel) OR Hydrogel) OR Gelatin) OR Ointment). The bibliographies of studies that fulfilled eligibility criteria were also examined to find articles not present in our initial search. This study followed the guidelines outlined in the preferred reporting items for systematic reviews and meta-analyses (PRISMA).

Selection Criteria

Eligibility criteria included studies reporting data on the use of a topical approach to delivering targeted therapies in the treatment of lymphedema. Therefore, we excluded papers that investigated other routes of therapy or those which applied to cases of edema not caused by post-surgical lymphedema. Abstracts, presentations, reviews, and meta-analyses were also excluded.

Data Extraction and Processing

Extracted data included the year, country, type of study, lymphedema model, substance/procedure, and treatment outcome. Data extraction from articles, tables, and figures was performed by two reviewers (D.B and M.T.H), with the accuracy of data entry confirmed by an additional reviewer (A.J.F).

Results

Study Characteristics

**Figure 1:**
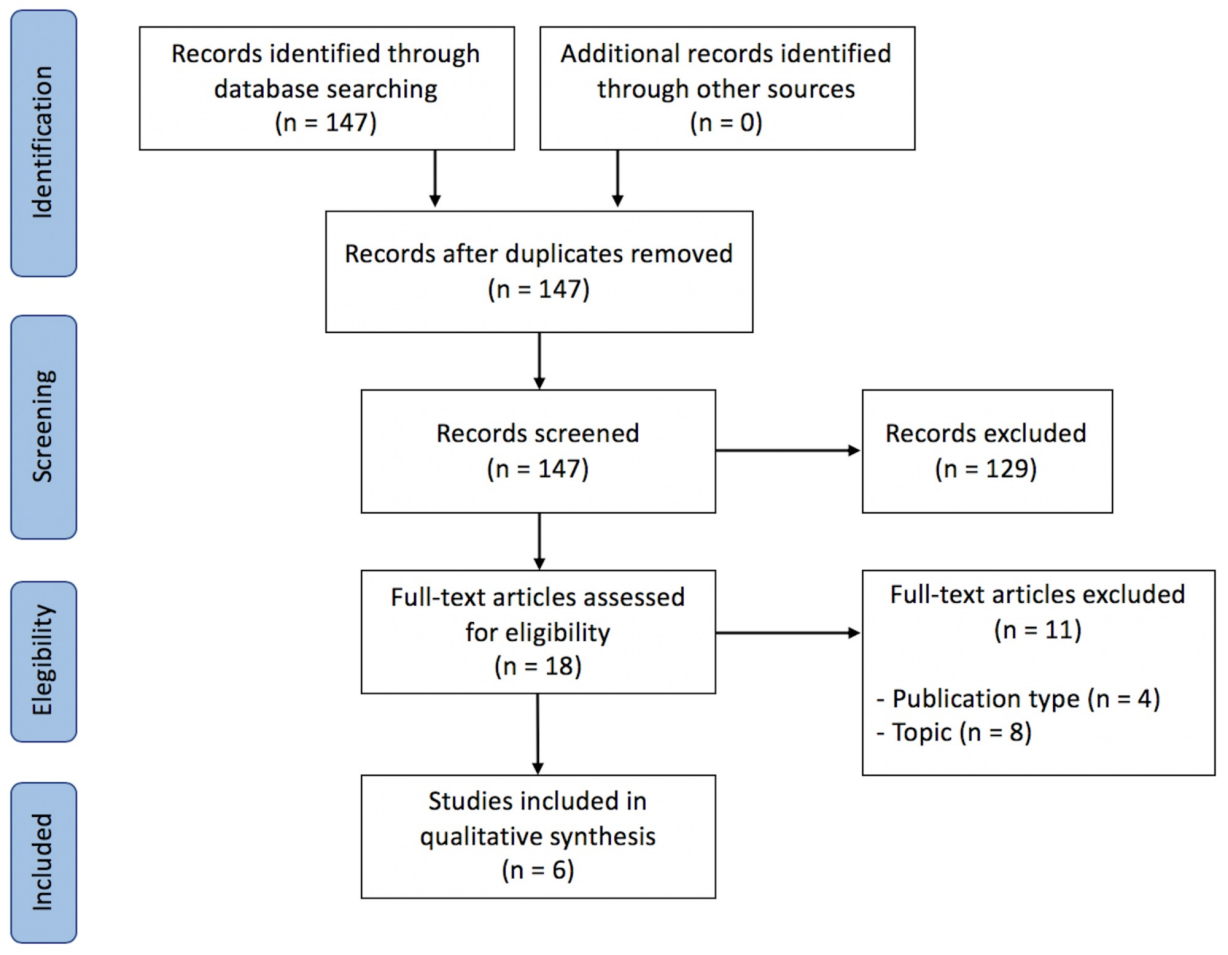
Preferred reporting items for systematic reviews and meta-analyses (PRISMA) diagram

Of the 147 papers found in the literature, six studies fulfilled the eligibility criteria (Figure 1; Table 1). In 1993, Casley-Smith et al. published a study on the use of a topical approach to delivering targeted therapies in the treatment of lymphedema [13]. It has been followed by other experimental studies conducted on small animals (mice). Interestingly, many have proposed different methods of therapy/procedures, which can be clustered into two groups: 1) induction of lymphangiogenesis [vascular endothelial growth factor C (VEGF-C) hydrogel or fibroblast growth factor] [14-16]; and 2) modulation of inflammation (tacrolimus, topical collagen gel, or troxerutin-phosphatidylcholine) [8,13,17].

**Table 1:** Summary of the study findings VEGF: vascular endothelial growth factor; ADSC: adipose-derived stem cell; bFGF: basic fibroblast growth factor; ESWT: extracorporeal shock wave therapy

Author	Year	Country	Study type	Model	Mechanism	Topical substance/procedure	Additional substance/procedure	Positive outcome
Gardenier et al. [8]	2017	US	Experimental	Mice	Anti-inflammation	Tacrolimus		Yes
Onishi et al. [16]	2014	Japan	Experimental	Mice	Lymphangiogenesis	bFGF		Yes
Kim et al. [14]	2013	South Korea	Experimental	Mice	Lymphangiogenesis	VEGF-C hydrogel	ESWT	Yes
Hwang et al. [15]	2011	South Korea	Experimental	Mice	Lymphangiogenesis	VEGF-C hydrogel	ADSC	Yes
Clavin et al. [17]	2008	US	Experimental	Mice	Anti-inflammation	Topical collagen gel		Yes
Casley-Smith et al. [13]	1993	Australia	Experimental	Mice	Anti-inflammation	Troxerutin-phosphatidylcholine complex in liposomal-like microdispersion		Yes

Induction of Lymphangiogenesis

Induction of lymphangiogenesis via topical therapy was investigated in three experimental studies [14-16]. Two studies evaluated the effect of a topical VEGF-C hydrogel in the treatment of lymphedema. Hwang et al. experimented with a mice lymphedema model to assess the lymphangiogenic potential of VEGF-C hydrogel with or without adipose-derived stem cells (ADSC) [15]. They observed that mice treated with both ADSC and VEGF-C hydrogel had greater edema reduction and lymphangiogenesis compared to mice treated with either ADSC or VEGF-C hydrogel alone [15]. Kim et al. experimented with mice models with lymphedema to evaluate the association of VEGF-C hydrogel with extracorporeal shock wave therapy (ESWT) [14]. They noted that, compared to the control group, this therapy promoted lymphangiogenesis [an increase in positive lymphatic vessel endothelial hyaluronan receptor 1 (LYVE-1) vessels, and expression of VEGF-C and VEGF receptor-3] and a decrease in edema [14]. The effect of topical basic fibroblast growth factor (bFGF) in lymphedema treatment was also investigated. Onishi et al. conducted a study on mice lymphedema models to evaluate the lymphangiogenic response to daily topical bFGF [16]. Their results showed that mice treated with bFGF had an improvement in lymphedema (smaller edema volume and earlier decrease in the intensity of indocyanine green fluorescence) and greater lymphangiogenesis (increased lymphatic vessels density and expression of VEGF-C) compared to the control group [16].

Modulation of Inflammation

Modulation of inflammation via topical therapy was proposed in two experimental studies [8,17]. Gardenier et al. conducted a study on mice lymphedema models to evaluate the effect of topical tacrolimus [US Food and Drug Administration (FDA)-approved immunosuppressive drug, anti-T-cell] [8]. They noticed a reduction in edema, tissue fibrosis, T-cell infiltration, and greater lymphatic function (increase in lymphatic vessel collaterals, vessel contraction, and reduced dermal backflow). This led them to postulate that topical tacrolimus had the potential to both prevent and treat lymphedema [8]. Clavin et al. experimented on mice lymphedema models to investigate the effect of transforming growth factor β1 (TGF-β1; regulator of tissue fibrosis/scarring) suppression by repairing wounds using a topical collagen gel [17]. Compared to controls, mice treated with collagen gel had increased proliferation of lymphatic endothelial cells (LECs), lower expression of TGF-β1, and lower fibrosis [17]. Casley-Smith et al. [13] conducted a study on a mice lymphedema model to assess the effect of topical application of liposomal troxerutin and phosphatidylcholine (anti-oxidative, anti-inflammatory, and anti-thrombolytic). They noted that the mice that received this therapy had 75% less edema (mean: 40%) and a decrease in edema compared to the control group [13].

Discussion

To the best of our knowledge, our study is the first systematic review to investigate the use of topical approaches to delivering targeted therapies in the treatment of lymphedema. In this study, we demonstrated that all of the studies conducted so far on the topic were experiments on small animals (mice) using the rationale to promote lymphangiogenesis or modulate tissue inflammation. Moreover, all studies presented positive outcomes, demonstrating that a topical approach is indeed a promising route of delivery for both growth factors and anti-inflammatory agents in the treatment of lymphedema. The studies reviewed the use of different treatments and there was no consistency between study protocols.

The idea of a simple topical intervention to treat lymphedema is highly attractive because of the small risk of systemic toxicity and the convenience of patient self-application. Gardenier et al., who studied the effects of topical tacrolimus in a small animal lymphedema model, pointed out that one of the main advantages of their proposed treatment is that it is already FDA-approved for other dermatologic diseases [8].

This study has limitations characteristic of systematic reviews, including the likelihood of bias in analyzing the data reported in each article. However, our review summarizes relevant scientific knowledge regarding the use of a topical approach to delivering targeted therapies in the treatment of lymphedema, which can contribute to future studies. Although current data demonstrate positive outcomes for the use of a topical approach in mice, human studies utilizing targeted topical therapies have not been performed. Therefore, we advocate for further studies to assess the efficacy and safety of topical therapy on large animal lymphedema models involving clinical trials or carefully designed studies that would look into promoting off-label use of already-used targeted topical therapies in the future.

## Conclusions

The scientific evidence that we presented for the use of a topical approach to delivering targeted therapy in the treatment of lymphedema is encouraging. The topical approach is able to effectively deliver growth factors and anti-inflammatory agents with positive effects as measured in mice. However, studies were conducted under heterogeneous protocols, and the safe application of this approach in humans has not been assessed. We believe that further studies are required to analyze and confirm the benefits and safety of targeted topical therapy on patients with lymphedema.
